# Differential expression of angiogenesis-related genes ‘VEGF’ and ‘angiopoietin-1’ in metastatic and EMAST-positive colorectal cancer patients

**DOI:** 10.1038/s41598-024-61000-x

**Published:** 2024-05-08

**Authors:** Amir Torshizi Esfahani, Somayeh Mohammadpour, Pooya Jalali, Alireza Yaghoobi, Raana Karimpour, Soha Torkamani, Ali Pardakhtchi, Zahra Salehi, Ehsan Nazemalhosseini-Mojarad

**Affiliations:** 1 Basic and Molecular Epidemiology of Gastrointestinal Disorders Research Center, Research Institute for Gastroenterology and Liver Diseases, Shahid Beheshti University of Medical Sciences, Tehran, Iran; 2 Gastroenterology and Liver Diseases Research Center, Research Institute for Gastroenterology and Liver Diseases, Shahid Beheshti University of Medical Sciences, Tehran, Iran; 3 School of Medicine, Shahid Beheshti University of Medical Sciences, Tehran, Iran; 4 Department of Cellular and Molecular Biology, Faculty of Advanced Science and Technology, Tehran Medical Sciences, Islamic Azad University, Tehran, Iran; 5 Hematology, Oncology and Stem Cell Transplantation Research Center, Tehran University of Medical Sciences, Tehran, Iran; 6 Research Institute for Oncology, Hematology and Cell Therapy, Tehran University of Medical Sciences, Tehran, Iran; 7 Present Address: Department of Surgery, Leiden University Medical Center, Leiden, Netherlands

**Keywords:** Colorectal cancer, Angiogenesis, Elevated microsatellite alteration at selected tetranucleotide repeats, EMAST, Metastasis, Cancer, Computational biology and bioinformatics, Molecular biology, Biomarkers, Gastroenterology, Medical research, Molecular medicine, Oncology

## Abstract

Abnormal angiogenesis leads to tumor progression and metastasis in colorectal cancer (CRC). This study aimed to elucidate the association between angiogenesis-related genes, including VEGF-A, ANGPT-1, and ANGPT-2 with both metastatic and microsatellite alterations at selected tetranucleotide repeats (EMAST) subtypes of CRC. We conducted a thorough assessment of the ANGPT-1, ANGPT-2, and VEGF-A gene expression utilizing publicly available RNA sequencing and microarray datasets. Then, the experimental validation was performed in 122 CRC patients, considering their disease metastasis and EMAST^+/−^ profile by using reverse transcription polymerase chain reaction (RT-PCR). Subsequently, a competing endogenous RNA (ceRNA) network associated with these angiogenesis-related genes was constructed and analyzed. The expression level of VEGF-A and ANGPT-2 genes were significantly higher in tumor tissues as compared with normal adjacent tissues (*P*-value < 0.001). Nevertheless, ANGPT-1 had a significantly lower expression in tumor samples than in normal colon tissue (*P*-value < 0.01). We identified a significantly increased VEGF-A (*P*-value = 0.002) and decreased ANGPT-1 (*P*-value = 0.04) expression in EMAST^+^ colorectal tumors. Regarding metastasis, a significantly increased VEGF-A and ANGPT-2 expression (*P*-value = 0.001) and decreased ANGPT-1 expression (*P*-value < 0.05) were established in metastatic CRC patients. Remarkably, co-expression analysis also showed a strong correlation between ANGPT-2 and VEGF-A gene expressions. The ceRNA network was constructed by ANGPT-1, ANGPT-2, VEGF-A, and experimentally validated miRNAs (*hsa-miR-190a-3p, hsa-miR-374c-5p, hsa-miR-452-5p,* and *hsa-miR-889-3p*), lncRNAs (*AFAP1-AS1, KCNQ1OT1* and *MALAT1*), and TFs (*Sp1, E2F1,* and *STAT3*). Network analysis revealed that colorectal cancer is amongst the 82 significant pathways. We demonstrated a significant differential expression of VEGF-A and ANGPT-1 in colorectal cancer patients exhibiting the EMAST^+^ phenotype. This finding provides novel insights into the molecular pathogenesis of colorectal cancer, specifically in EMAST subtypes. Yet, the generalization of in silico findings to EMAST^+^ colorectal cancer warrants future experimental investigations. In the end, this study proposes that the EMAST biomarker could serve as an additional perspective on CMS4 biology which is well-defined by activated angiogenesis and worse overall survival.

## Introduction

Angiogenesis is a naturally occurring and vital physiological process for the growth and development of normal tissue cells during embryonic development and later, which is regulated by a fine balance between a range of stimulatory factors promoting the growth of new blood vessels and inhibitory signals blocking the uncontrolled expansion of new blood vessels^1,2^. However, under certain circumstances, the effect of activators surpasses those of the inhibitors, and this phenomenon can lead to certain aggressive diseases, including tumor progression and metastasis^3^. In other words, angiogenesis plays a prominent role in tumor growth and proliferation and is approved to be a prerequisite for metastatic dissemination of colorectal tumors^4,5^.

Angiogenesis is assessed by multiple angiogenic factors, and among them, the most prominent tumor angiogenesis regulator is vascular endothelial growth factor (VEGF) and its receptors (VEGFR-1, VEGFR-2, and VEGFR-3)^6^. VEGF is a signaling glycoprotein identified as a crucial stimulator of angiogenesis by which colon tumors’ growth is promoted via increased nutrient provision^7^. Moreover, the VEGF-induced signaling pathway is fundamental for tumor progression and participates significantly in the metastasis of colorectal cancer^8^. In this regard, a significant correlation between VEGF overexpression and poor prognosis has been outlined in CRC patients^9^. Besides VEGF, Angiopoietins (ANGPT-1 and ANGPT-2) and their receptor, Tie-2, are shown as other determining elements involved in the induction of angiogenesis^4^. Angiopoietin-1 (ANGPT-1) has been termed the chief ligand that induces activation of the Tie-2 receptor via phosphorylation and, therefore, promotes endothelial cell survival and contributes to vessel stabilization^10^. Furthermore, previous investigations seem to support the idea that unlike VEGF and ANGPT-2, the expression level of ANGPT-1 is significantly lower in colon cancer cells compared to normal colonic epithelium, and transfecting human colon carcinoma cell line with ANGPT-1 inhibited the tumor cell proliferation and growth^11,12^. Angiopoietin-2 (ANGPT-2) performs an antagonistic function towards ANGPT-1 by preventing the Tie-2 receptor’s activation, which results in vessels being structurally and functionally fragile and unstable^11,13^. This incident is an essential and inseparable step in the induction of abnormal angiogenesis in several malignancies, including CRC. Therefore, many studies have suggested that overexpression of angiogenesis-related genes, namely, VEGF and ANGPT-2, makes them useful prognostic biomarkers for patients with advanced colorectal carcinoma^9,10^.

A growing body of research shows that there are other potent prognostic biomarkers for the management of patients with CRC, including microsatellite instability (MSI). Microsatellite instability is characterized by a deficient DNA Mismatch Repair (MMR) system^14^. Apart from the classic form of MSI, there exists another variant known as elevated microsatellite alterations at selected tetranucleotide repeats (EMAST), which exclusively occurs at tetranucleotide microsatellite DNA sequences, delineating its distinct nature^15^. EMAST is a genetic variation present in over thirty percent of CRC patients^16^. Furthermore, the EMAST biomarker has recently been proposed to significantly correlate with tumor metastasis and poor prognosis^17^. In this regard, our previous study investigated the precise association between EMAST phenomenon and Snail as a gene related to the epithelial-to-mesenchymal transition (EMT) process in CRC patients^18^.

Here, we aimed to elucidate the association between angiogenesis-related genes with both tumor metastasis and EMAST subtypes of colorectal cancer. Hence, we provided a comprehensive examination of ANGPT-1, ANGPT-2, and VEGF-A genes expression in CRC patients. We marked differences in the mean expression values of each gene in line with clinicopathological features such as metastatic state and EMAST biomarker and introduced novel biomarkers for evaluating cancer progression.

## Materials and methods

**Figure 1:**
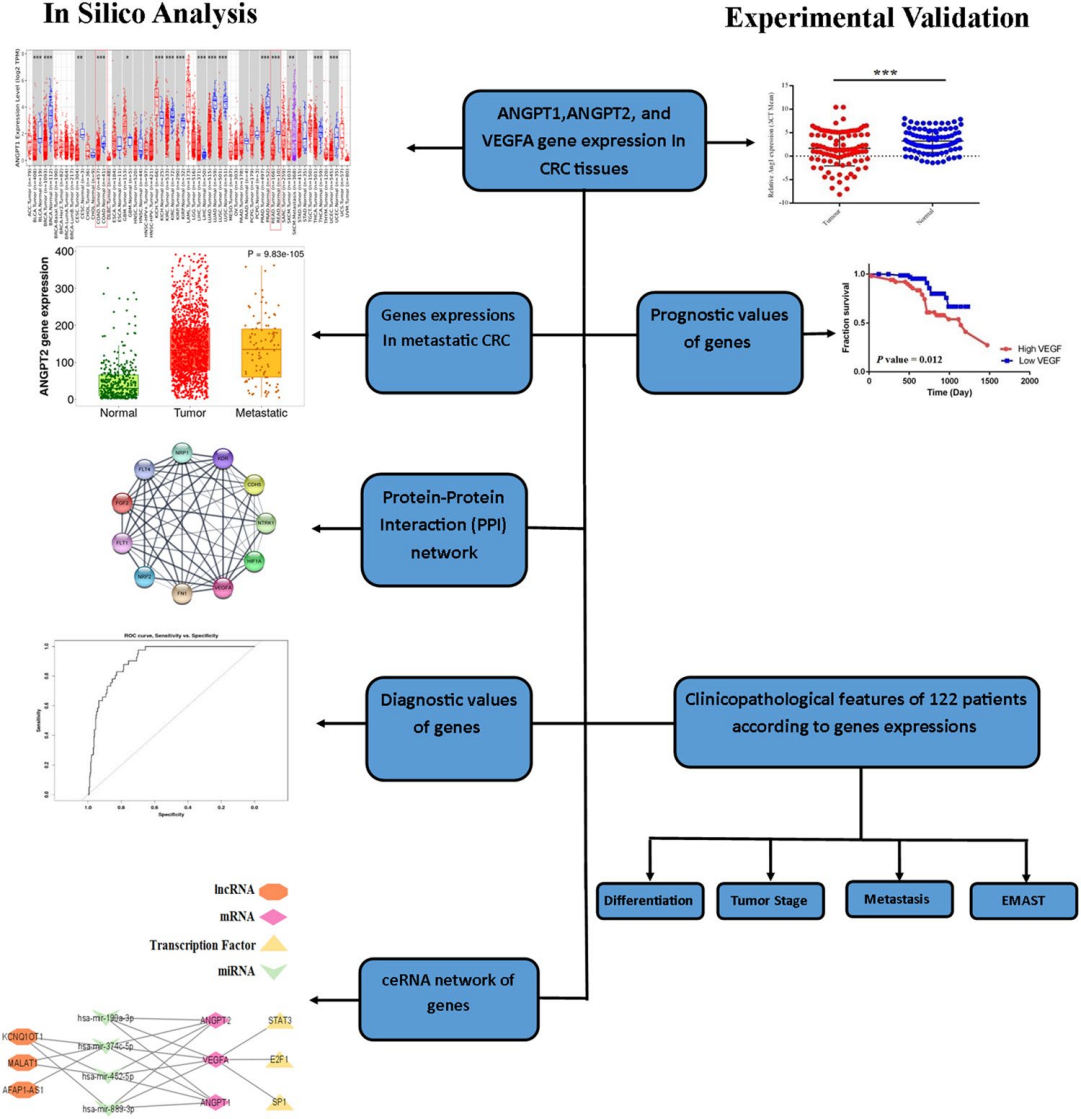
The Study flowchart. We used different bioinformatics analysis to determine the correlation of the angiogenic ANGPT-1, ANGPT2, and VEGF-A factors with colon and rectum cancer progression. The results were further validated experimentally by running Real-time PCR on CRC patients’ tissue samples. We further experimentally evaluated the association of ANGPT-1, ANGPT-2, and VEGF-A with EMAST^+^ patient samples. CRC: Colorectal Cancer, ceRNA: Competitive Endogenous RNA.

We carried out a comprehensive evaluation of ANGPT-1, ANGPT-2, and VEGF-A genes' expression in patients with colorectal cancer, particularly metastatic and EMAST subtypes (referred to mCRC and EMAST, respectively). We also conducted bioinformatics analysis through publicly available RNA sequencing and microarray datasets. Then, the gene regulatory network of angiopoietin-associated genes, including experimentally validated transcription factors, miRNAs, and lncRNAs sponging selected miRNAs were identified and ceRNA network was constructed. Finally, we examined the differential expression levels of the genes via RT-PCR (Fig. 1).

### Data collection

TIMER2 (Tumor Immune Estimation Resource version 2, http://timer.cistrome.org/)^19^ was used to evaluate the expression of ANGPT-1, ANGPT-2, and VEGF-A in tumor tissue from COAD and READ and in adjacent normal tissues of the TCGA project. Also, the Gene Expression database of Normal and Tumor tissues 2 (GENT2) (http://gent2.appex.kr/gent2/)^20^ was utilized to define the genes expression across microarray datasets. To collect gene expression datasets related to metastatic CRC, TNMplot database (https://www.tnmplot.com/) was utilized based on Gene Chip data^21^.

### Diagnostic value of ANGPT-1, ANGPT-2, and VEGF-A in CRC

To study the diagnostic value of ANGPT-1, ANGPT-2, and VEGF-A, gene expression profiles of selected genes in COAD and READ were obtained from OncoDB database (https://oncodb.org/)^22^. Then, we evaluated the diagnostic value of selected genes in COAD and READ and their adjacent normal tissues utilizing https://analysistools.cancer.gov/biomarkerTools.

### Co-expression analysis and PPI network

In this study, the top 50 genes positively and negatively correlated with ANGPT-1, ANGPT-2, and VEGF-A in colorectal cancer were obtained through LinkedOmics^23^. The protein–protein interaction (PPI) network involving these selected genes was constructed using the STRING database (https://string-db.org) and visualized using Cytoscape software (version 3.9.1; https://cytoscape.org). Additionally, top genes exhibiting similar expression patterns to ANGPT-1, ANGPT-2, and VEGF-A genes were sourced from the GEPIA2 database^24^. An intersection analysis was conducted to identify common proteins interacting with these genes and the top 100 similarly expressed genes.

### CeRNA network construction

This research recognizes miRNAs as regulators of post-transcriptional gene expression^25^, while Transcription Factors (TFs) play a pivotal role in influencing transcription rates during the pre-transcriptional stage^26^. miRNet (https://www.mirnet.ca/miRNet/home.xhtml)^27^ and TFcancer (http://lcbb.swjtu.edu.cn/tfcancer/gesearch.html)^28^ databases were used to determine the impact of TFs on the expression of selected genes in COAD and READ. The miRNAs targeting ANGPT-1, ANGPT-2, and VEGF-A genes were identified using mirDB (https://mirdb.org/mirdb/index.html)^29^ and DIANA-microT (https://dianalab.e-ce.uth.gr/microt_webserver/#/interactions)^30^. In addition, identified miRNAs were analysed in CancerMIRNome database (http://bioinfo.jialab-ucr.org/CancerMIRNome/) in order to identify experimentally validated miRNAs. Furthermore, circRNAs regulating these validated miRNAs were sourced from the circBank database (http://www.circbank.cn/)^31^, with a focus on those with a tot score exceeding 1000. Additionally, lncRNAs targeting selected miRNAs were identified utilizing miRNet and LncTarD2 (https://lnctard.bio-database.com/)^32^ databases. Finally, A competitive endogenous RNA (ceRNA) network encompassing ANGPT-1, ANGPT-2, and VEGF-A genes, experimentally validated TFs influencing these genes, experimentally validated miRNAs targeting selected genes, and experimentally validated lncRNAs targeting the validated miRNAs was constructed using Cytoscape software^33^. Furthermore, NcPath database was utilized to enrich the ceRNA network (http://ncpath.pianlab.cn/#/Home)^34^.

### Participants

Precisely 122 CRC patients who experienced surgery at Taleghani and Shohada Tajrish Hospitals of Shahid Beheshti University of Medical Sciences were recruited for this study. Our former studies assessed the EMAST status in the CRC patients^35^. Eligible individuals included in the study met the following criteria for inclusion: (a) patients diagnosed with adenocarcinoma based on histological examination, (b) availability of pertinent clinical data and pathology reports, and (c) patients who underwent adjuvant chemotherapy utilizing either 5-FU or no treatment. Furthermore, the expression levels of VEGF-A, ANGPT-1, and ANGPT-2 were evaluated in formalin-fixed paraffin-embedded (FFPE) archived tumor samples and adjacent normal colorectal tissues, and their correlation with CRC metastasis, CRC patients’ survival, and EMAST biomarker was determined.

### EMAST evaluation

As mentioned, we conducted an experiment evaluating EMAST phenotype among these patients in our previous study^36^. Concisely, the FFPE DNA extraction kit manufactured by QIAGEN GmbH (QIAGEN GmbH, Germany) was applied to extract DNA from tumor and normal adjacent FFPE tissues (NAT). A panel using five tetranucleotide markers, namely D9S242, MYCL1, D8S321, D20S82, and D20S85 was used for EMAST analysis. PCR optimization was carried out by using previously designed primers^37^.

Moreover, QIAxcel capillary electrophoresis, High-resolution cartridge, 25–500 nucleotide molecular markers, and 15–156 nucleotide align marker was employed for detachment of segments produced by PCR, and at the same time to provide a valid comparison between the microsatellite instability status in both tumor and NAT specimens of each patient^37^. Samples were classified as EMAST positive (EMAST^+^) when at least two of five markers showed a different pattern in tumor cells than normal. If just one or none of the microsatellite locus demonstrated a distinct pattern in tumor cells rather than normal, samples were defined as EMAST negative (EMAST^−^).

### RNA isolation and cDNA synthesis

RNA was extracted from CRC and normal adjacent FFPE (NAT) samples via the RNeasy® FFPE kit (QIAGEN, Germany) as instructed by the manufacturer. RNA concentration and quality were measured using a Nano-Drop ND-1000 Spectrophotometer (Thermo Scientific, USA). Afterward, the isolated RNA went through a reverse transcription procedure using Prime Script-RT Master Mix (Takara Bio Inc., Otsu, Japan), following the manufacturer's recommended protocol. Once the complementary DNA (cDNA) had been synthesized, they were stored at − 20 °C.

### Quantitative reverse transcriptase PCR

The expression levels of angiogenic mediators were quantified with quantitative reverse transcriptase PCR (RT-PCR) using Light Cycler ABI 7500 Real-time PCR system and Maxima® SYBER Green/Rox with MicroAMP Optical 96-well reaction (Applied Biosystems, USA) pellet. The specific primers for VEGF-A, ANGPT-1, ANGPT-2, and β-actin were designed (Table 1). The PCR amplification profile was run as follows: 30 s initial denaturation at 95 °C followed by 45 cycles of cycling stage: 95 °C for 5 s (Denaturation), 60 °C for 34 s (Annealing) and finally 72 °C for 30 s (Extension). Each sample was measured at least in duplicate. A total reaction volume of 20μl containing 0.4 μl of each primer, 10μl Maxima SYBER Green/Rox and 5μl cDNA was used for Real-time PCR analysis. Gene expression values for all specimens were analyzed using the 2^−∆∆CT^ (Threshold cycle) approach, and these data were normalized to the expression of the housekeeping gene, β-actin, forward and reverse primers. It is noteworthy that the efficacy of each primer was calculated using LinReg Software (version: 2017.1). The relative quantification (RQ) values were applied in statistical analysis.

**Table 1:** List of primer sequences used for RT-PCR analysis in this study.

Gene	Primers	Length (bp)
ANGPT-1	**Forward:** CATGTTAACAGGAGGATGGTGG	74
	**Reverse:** GTCCCGCAGTATAGAACATTCC	
ANGPT-2	**Forward:** CAAACTCAGCTAAGGACCCCAC	89
	**Reverse:** CGTGGTGTGTCCTGATTTGAATAC	
VEGF-A	**Forward:** AGTGGTCCCAGGCTGCAC	70
	**Reverse:** TCCATGAACTTCACCACTTCGT	
β-actin	**Forward:** CACCATTGGCAATGAGCGGTTC	135
	**Reverse:** AGGTCTTTGCGGATGTCCACGT	

### Statistical analysis

All resulting data were statistically executed using GraphPad Prism 6.01 (GraphPad Software Inc., San Diego, USA) coupled with SPSS 16.0 (SPSS Inc., Chicago, IL). The chi-square method was applied to assess the disparities among variables. Shapiro–Wilk test was used to assess whether our data follow normal distribution pattern or not. Accordingly, the information consistent with normal distribution was stated as mean ± standard deflection and examined with independent sample t-test. Otherwise, Mann–Whitney U test was used to assess whether the differences were statistically significant. Kaplan–Meier curves for overall survival were made using GraphPad prism software. In addition, a log-rank test was used to approach the contrast through survival curve groups. In the entire examination, *P*-values of less than 0.05 were affirmed statistically significant.

### Ethics approval and consent to participate

The study adhered to the ethical guidelines outlined in the Declaration of Helsinki, including obtaining written consent from all human research participants. All procedures were conducted under the oversight of the Ethics Committee of the Gastroenterology and Liver Disease Research Institute of Shahid Beheshti University of Medical Sciences (No: IR.SBMU.RIGLD.REC.1395.136) in accordance with the university’s policies on medical and research ethics. Informed consent was obtained from all participants included in the study.

## Results

### ANGPT-1, ANGPT-2, and VEGF-A expression in colorectal cancer datasets.

**Figure 2:**
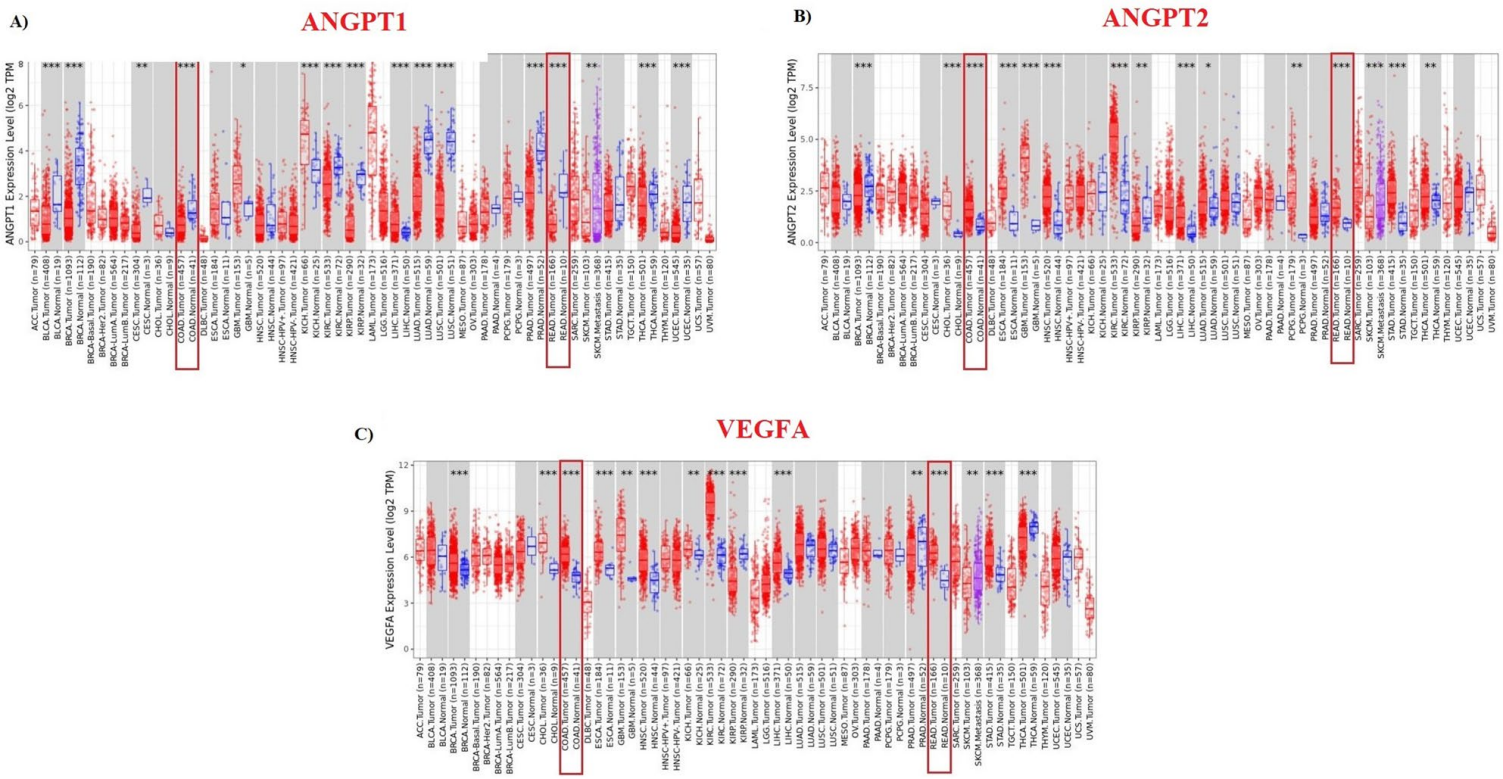
Gene expression profiles of ANGPT1, ANGPT2, and VEGFA based on RNASeq obtained from TIMER2 database. (**A**) ANGPT-1 is down-regulated in COAD and READ, however, ANGPT-2 (**B**) and VEGF-A (**C**) were upregulated in COAD and READ compared to normal tissues.

**Figure 3:**
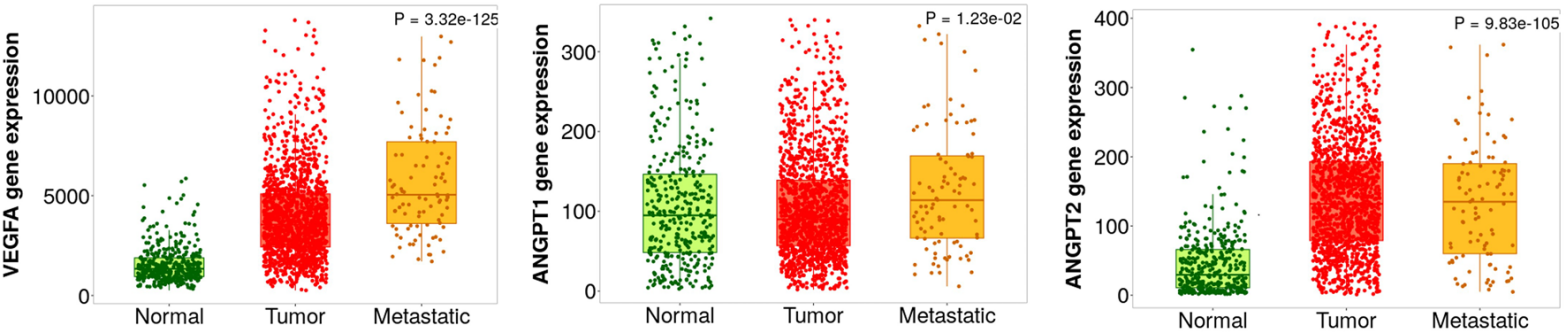
VEGF-A, ANGPT-1, and ANGPT-2 gene expressions in normal, tumor, and metastatic CRC tissues extracted form TNMplot database. All selected genes were significantly upregulated in metastatic colon tissues compared to normal tissues.

Based on data retrieved by TCGA project, ANGPT-1 was significantly downregulated (*P*-value < 0.001) (Fig. 2A), while ANGPT-2 and VEGF-A were significantly upregulated in COAD and READ tissues compare to normal tissues (*P*-value < 0.001), respectively (Fig. 2B, C). Microarray data analysis via GENT2 database was also consistent to TCGA data (Supplementary File 1). To evaluate the gene expression profile in metastatic colorectal cancer, TNMplot was utilized. Notably all selected genes were significantly upregulated in metastatic colon tissues compared to normal tissues (*P*-value < 0.05) (Fig. 3).

### Diagnostic values of ANGPT1, ANGPT2, and VEGF-A

Multiple studies have shown the diagnostic value of angiogenesis-related genes including S100A4, RBP4, and THBS2 in colorectal cancer^38,39^. Using RNA sequencing data obtained from OncoDB, ANGPT-2 (AUC = 90.8%) and VEGF-A (AUC = 95.1%) in COAD and VEGF-A in READ (AUC = 97.3%) had AUC > 90% and can be diagnostic factors for colorectal cancer (Supplementary File 1).

### PPI and CO-expression Network shows VEGF-A and ANGPT-2 are co-expressed

**Figure 4:**
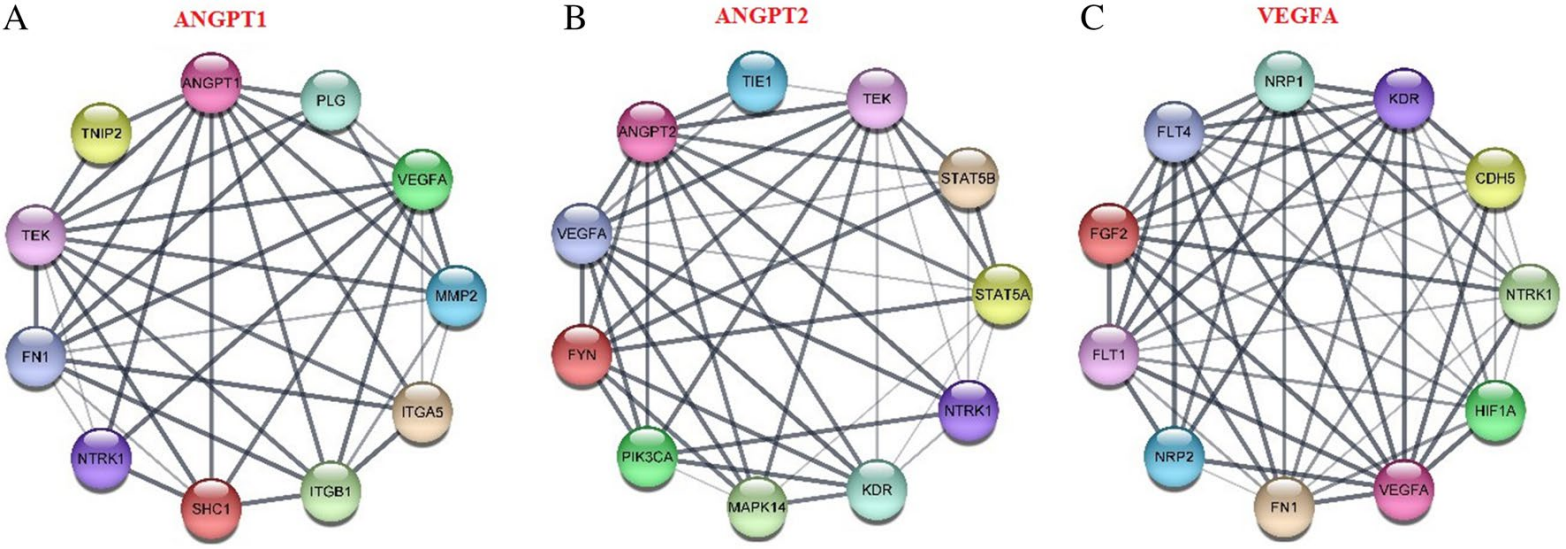
The Protein–protein interaction networks of ANGPT-1, ANGPT-2, and VEGF-A. PPI networks for ANGPT-1, ANGPT-2, and VEGF-A contained 11 nodes and 38 edges (**A**), 11 nodes and 42 edges (**B**), and 11 nodes and 51 edges (**C**), respectively.

ANGPT-1, ANGPT-2, and VEGF-A co-expressed genes were evaluated using LinkedOmics, which provided the data from TCGA-COAD-READ cohort. The heatmap and volcano plot of the top 50 positively and negatively correlated genes were constructed (supplementary File 1). The top positively and negatively correlated genes with ANGPT-1, ANGPT-2 and VEGF-A were *OLFML1-COL4A1- GTPBP2* and *C6ORF136-ELMO3-FECH*, respectively. Furthermore, interacting genes were screened for protein–protein interactions (PPI) network construction using STRING database. PPI networks for ANGPT-1, ANGPT-2, and VEGF-A contained 11 nodes and 38 edges (Fig. 4A), 11 nodes and 42 edges (Fig. 4B), and 11 nodes and 51 edges (Fig. 4C), respectively. Besides, we performed an intersection analysis between interacted genes and the top 100 genes that have similar expression pattern with ANGPT-1, ANGPT-2, and VEGF-A obtained from GEPIA2. No genes identified as common gene between ANGPT-1 interacted and similar genes, however, VEGF-A and *KDR* were identified as common genes between ANGPT-2 interacted and similar genes. In addition, *FLT-1, FLT-2, NRB-1,* and *KDR* were common in VEGF-A interacted and similar genes (Supplementary File 1). There was a strong correlation between VEGF-A and ANGPT-2 and they co-express together. Since ANGPT-2 and VEGF-A are crucial regulators of vascular remodeling, co-targeting VEGF-A and ANGPT-2 in combination with chemotherapy in chemoresistant CRC xenograft models can be of paramount importance in future studies^40^.

### The ceRNA network construction

Based on DIANA-microT and miRDB, 18 miRNAs were found as common miRNAs targeting ANGPT-1, ANGPT-2, and VEGF-A genes. Remarkably, *hsa-miR-147b-5p* was found as the miRNA targeting the three angiogenic genes. Among 18 identified miRNAs, *hsa-miR-190a-3p, hsa-miR-374c-5p, hsa-miR-452-5p,* and *hsa-miR-889-3p* were experimentally validated in TCGA COAD or READ tissues based on CancerMIRNome database (Supplementary File 1).

By utilizing CircBank database, 19 circRNAs were identified sponging *hsa-miR-374c-5p*. No circRNAs were found having interaction with *hsa-miR-190a-3p, hsa-miR-452-5p,* and *hsa-miR-889-3p*. A total of 203 lncRNAs were found which were related to 8 miRNAs, among 18 selected miRNAs. Among them, 5 lncRNAs, including *CRNDE, AFAP1-AS1, KCNQ1OT1, MIR100HG,* and *MALAT1* were experimentally validated as a differentially expressed lncRNA based on LncTarD 2 (Supplementary Files 1, 2). *AFAP1-AS1, KCNQ1OT,* and *MALAT1* were found sponging identified validated miRNAs.

Using miRNet database, a total of 72 TFs were identified in relation to ANGPT-1, ANGPT-2, VEGF-A. Among identified TFs, *Sp1, E2F1,* and *STAT3* were experimentally validated in COAD-READ tissues based on TFcancer database (Supplementary Files 1, 2).

**Figure 5:**
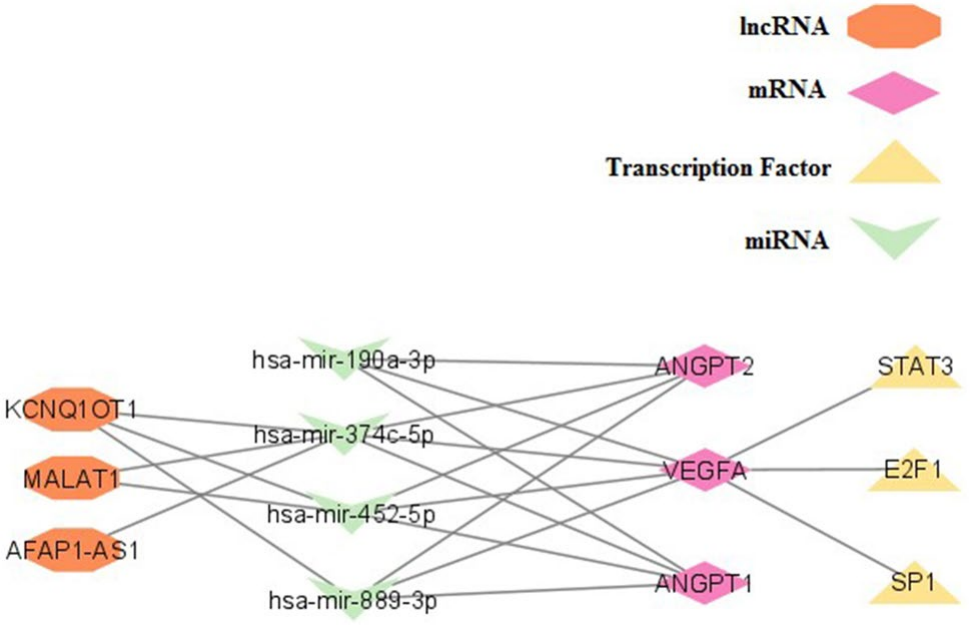
ANGPT-1, ANGPT-2, VEGF-A gene regulatory network. ceRNA network including ANGPT-1, ANGPT-2, VEGF-A, their validated experimentally validated Transcription factors, experimentally validated miRNAs targeting selected genes, and experimentally validated lncRNAs sponging selected miRNAs.

The ceRNA network, including ANGPT-1, ANGPT-2, VEGF-A, experimentally validated miRNAs (*hsa-miR-190a-3p, hsa-miR-374c-5p, hsa-miR-452-5p,* and *hsa-miR-889-3p*), lncRNAs (*AFAP1-AS1*, *KCNQ1OT1* and *MALAT1*), and TFs (*Sp1, E2F1,* and *STAT3*) was constructed. Based on network analysis, a total of 82 significant pathways were identified which MAPK signaling pathway, FoxO signaling pathway, mTOR signaling pathway, Cell cycle, Focal adhesion, and Colorectal cancer were notable significant pathways (Fig. 5).

### Altered gene expression of VEGF-A, ANGPT-1, and ANGPT-2 in colorectal cancer patients

**Figure 6:**
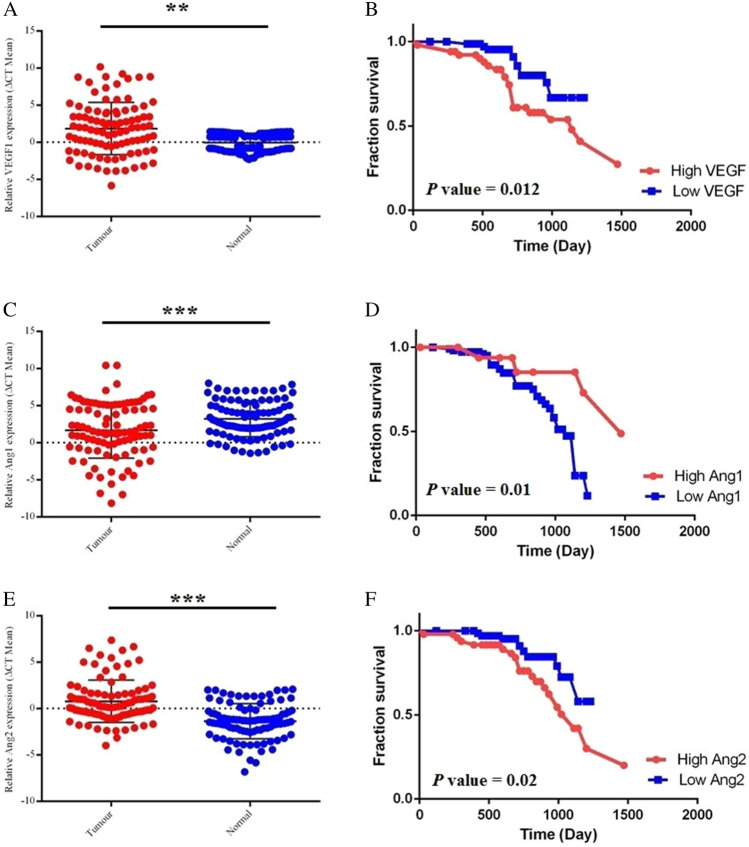
Experimentally gene expression profile and prognostic values of ANGPT-1, ANGPT-2, and VEGF-A in CRC patients. VEGF-A (**A**) and ANGPT-2 (**E**) were significantly upregulated, while ANGPT-1 (**C**) was significantly downregulated in CRC tissues compared to adjacent normal tissues. Also, downregulated ANGPT-1 (**D**) and upregulated ANGPT-2 (**F**) and VEGF-A (**B**) were significantly correlated with decreased overall survival of CRC patients.

To experimentally validate the differential expression of ANGPT-1, ANGPT-2, and VEGF-A genes, Real-time PCR assay was employed on the specimens of 122 CRC patients. As shown in Fig. 6, the expression level of VEGF-A and ANGPT-2 genes were significantly higher in tumor tissues as compared with normal adjacent tissues (*P*-value < 0.001). Nevertheless, ANGPT-1 had a significantly lower expression pattern in tumor samples than normal colon tissue (*P*-value = 0.003).

Further, the association between overall survival (OS) and Angiogenesis-related gene expression was also assessed. Thirty-nine patients had died within 50 months of follow-up (range, 8–82 months). The whole patient cohort's median overall survival (OS) time was 50 months, with a nominal 1-, 3- and 5-year survival of 96%, 88%, and 49%, respectively. Further, the resulting data shows a significant association between high expression of VEGF-A and ANGPT-2 and decreased OS (*P*-value = 0.012 and *P*-value = 0.01 respectively). Additionally, ANGPT-1 downregulation was significantly associated with poor overall survival (*P*-value = 0.02) (Fig. 6).

### Altered gene expression of VEGF-A and ANGPT-1 in EMAST^+^ colorectal cancer patients

The mean values of RQ for VEGF-A was discovered to be higher in patients with stage III/IV colorectal cancer (*P*-value = 0.001), but no statistically significant differential expression was identified between ANGPT-1 and ANGPT-2 expression and tumor stage. The expression level of all three angiogenesis-related genes was not significantly different in terms of tumor differentiation (*P*-value > 0.05).

Moreover, 43% of the CRC patients were detected with metastasis. Patients with metastatic CRC indicated a significantly higher mean value of RQ for VEGF-A and ANGPT-2 compared to non-metastatic CRC patients (*P*-value = 0.001). On the contrary, the expression level of ANGPT-1 was significantly lower in metastatic patients when compared with non-metastatic CRC patients (*P*-value < 0.05). Additionally, 40.2% of the patients were characterized as EMAST^+^ and 59.8% as EMAST^−^. The upregulation of VEGF-A and downregulation of ANGPT-1 was shown in EMAST^+^ phenotype (*P*-value = 0.002 and *P*-value = 0.04, respectively). However, there was no significant correlation between ANGPT-2 expression and EMAST phenotype. Table 2 provides data on marked differences in the mean values of RQ of each gene in line with clinicopathological features such as metastatic state and EMAST biomarker.

**Table 2:** Clinicopathological features of 122 patients according to VEGF-A, ANGPT-1, and ANGPT-2 expression.

Characteristics	N	VEGF-A 2^ΔΔCT^		ANGPT1 2^ΔΔCT^		ANGPT2 2^ΔΔCT^	
		Mean ± SD	*P*-value	Mean ± SD	*P*-value	Mean ± SD	*P*-value
Tumour stage							
I-II	70	9.52 ± 20.74	**0.001**	1.21 ± 2.31	0.53	8.68 ± 15.16	0.057
III-IV	52	21.84 ± 28.36		1.05 ± 2.03		12.02 ± 18.15	
Differentiation							
Well	40	13.28 ± 25.13	0.1	0.89 ± 2.01	0.07	10.23 ± 16.34	0.96
Moderate/ Poor	82	15.95 ± 25.23		1.26 ± 2.27		10.05 ± 16.69	
Metastasis							
Yes	52	21.44 ± 27.38	**0.001**	0.92 ± 1.67	**0.02**	13.65 ± 16.74	**0.001**
No	70	10.34 ± 22.36		4.31 ± 2.50		7.47 ± 15.95	
EMAST							
Positive	49	21 ± 27.15	**0.002**	0.93 ± 2.01	**0.04**	11.82 ± 17.89	0.19
negative	73	10.96 ± 22.92		5.46 ± 2.41		8.95 ± 15.53	

Significant values are in [bold].

## Discussion and future direction

Angiogenesis is a multistep mechanism necessary for CRC tumor development and progression, and it drives the metastasis of malignant tumor cells^41^. Numerous signaling pathways are associated with angiogenesis, including VEGF-A, ANGPT-1, and ANGPT-2, which significantly contribute to tumor growth and metastasis by providing oxygen, nutrients, and a safe microenvironment for tumor cells^42^. The rapid progress in identifying cancer biomarkers has shown promise in equipping precision medicine with the most appropriate treatment options based on the individual’s unique biomarker profile^43,44^. In this regard, genomic instability, particularly MSI, exhibits a pivotal role in tumor initiation and malignancies, and there is emerging evidence that this biomarker can be defined as a promising prognostic biomarker for CRC patients^45^. Previous studies on EMAST association with colorectal cancer similarly concluded a significant association between EMAST^+^ phenotype and advanced tumor stage in CRC^46,47^, and EMAST was previously reported to be involved in the metastatic spread of colorectal tumors and is associated with poor survival in patients with CRC^15^. Garcia et al. claimed that hypoxia induces genomic instability and is the link between EMAST^+^ phenotype and recurrent metastasis^48^. Since hypoxia is a major stimulator of angiogenesis, we can presume that angiogenesis is of paramount importance in EMAST^+^ cancer progression.

In our study, evaluating VEGF-A, ANGPT-1, and ANGPT-2 gene expression regarding clinicopathological features and also patient survival showed significantly increased expression levels of VEGF-A and ANGPT-2 in elevated tumor metastasis. Despite the significant positive correlation between VEGF-A and ANGPT-2 genes with metastasis, ANGPT-1 expression was significantly lower in patients with metastatic tumors. Previous investigations on the expression pattern of ANGPT-1 and ANGPT-2 in the plasma of patients with CRC indicated significantly higher expression levels of ANGPT-2 in patients with CRC, while the expression pattern of ANGPT-1 was not significantly different compared to healthy control. Moreover, the expression of ANGPT-2 was shown to be statistically upregulated in patients with stage IV tumors compared to patients with stage II tumors, while the expression of ANGPT-1 was unrelated to different colon cancer stages^49^. The present findings of our study are completely in line with the fact that blood vessel destabilization, caused by ANGPT-2 is a fundamental step in CRC aberrant angiogenesis. However, it is widely accepted that angiopoietins alone have limited potency, and they take part in the regulation of other angiogenic factors as well^50^. Like ANGPT-2, serum VEGF-A levels have been associated with the disease stage in CRC, with higher values being related to more advanced disease^51–53^. Vascular endothelial growth factor (VEGF) is secreted by multiple cell types, including cancer cells, and plays a major role in endothelial cell survival, growth, differentiation, and migration. As previously reported, VEGF-expressing CRCs possess increased growth and metastatic potential compared to tumors with baseline VEGF expression^51^. In a cohort of 103 patients with mCRC, a high serum VEGF-A level was demonstrated as a valuable predictive biomarker of liver and lung metastasis^54^. Therefore, compelling evidence implies a prognostic value of highly-expressed VEGF in CRC, and its correlation with poor prognosis^55,56^. Our findings strongly correlate with these former studies and further throw light on defining VEGF as a potential prognostic biomarker of tumor behavior. Another significant finding of the present study was the close significant correlation between overexpression of VEGF and ANGPT-2 and poor overall survival. Other studies have also observed a significant correlation between ANGPT-2 gene expression and poor overall survival as well^41,42,57^.

In the current study, we identified a significant increased VEGF-A and decreased ANGPT-1 gene expression in EMAST^+^ phenotype in colorectal tumors. This could potentially be the primary underlying mechanism contributing to the increased metastasis and poor prognosis observed in EMAST^+^ colorectal cancer patients in our previous research^36^, which revealed a higher prevalence of microsatellite instability-low (MSI-L) phenotype among patients diagnosed with Stage III and IV CRCs, compared to MSI-high (MSI-H) and microsatellite stable (MSS) tumors. On the other hand, in that study, we observed that EMAST^+^ status was relatively frequent in MSI-L CRCs, and MSI-L/EMAST^+^ was also identified as an unfavorable prognostic marker in CRC patients, associated with increased metastasis and lower overall survival rates^36^.

When determining the genes with similar expressional patterns as ANGPT-1, ANGPT-2, and VEGF-A, we identified a strong correlation between VEGF-A and ANGPT-2 as they co-express together. Moreover, VEGF-A and ANGPT-2 commonly had *KDR* as an overlapped gene with similar expression pattern. Meanwhile, VEGF-A has been demonstrated to regulate angiogenesis and vascular permeability by activating VEGFR-2 (*KDR*)^6^. Since ANGPT-2 and VEGF-A are crucial regulators of vascular remodeling, future studies may focus on co-targeting VEGF-A and ANGPT-2 in combination with chemotherapy in chemo-resistant CRC xenograft models^40^.

The regulatory networks involved in VEGF-A, ANGPT-1, and ANGPT-2 differential expression, including non-coding RNAs (ncRNAs) and transcription factors (TFs) were also investigated. Consequently, we constructed the ceRNA network with three mRNAs, four miRNAs, three lncRNAs, and three TFs. Accordingly, 4 experimentally validated miRNAs were used for ceRNA network construction. One of them, *hsa-miR-374c-5p,* was previously shown to upregulate in response to 5-Fluorouracil and starvation in the human colorectal adenocarcinoma cell line. This expression alteration was associated with increased autophagy of these cells and may be the mechanism of 5-Fluorouracil action in CRC patients as well^58^. Likewise, other studies further suggested *hsa-miR-190a-3p* and *hsa-miR-452-5p* as potential CRC diagnostic biomarkers^59–61^. Hence, investigating the role of ncRNAs and interpreting their interactions within ceRNA network could provide a deep insight into developing novel therapeutic targets and biomarkers, presuming that these networks are also active within EMAST^+^ phenotype. However, more experimental studies are required to ascertain the ncRNAs' roles in EMAST^+^ CRC angiogenesis and metastasis potential. In this study, after bioinformatically enriching the obtained ceRNA network, colorectal cancer was revealed as one of the notable significant pathways.

Transcription factors are also regulators of gene expression and can play essential roles in cancer progression and metastasis. Accordingly, using bioinformatics analysis, we found Sp1, E2F1, and STAT3 as the only experimentally validated TFs in COAD-READ registered tissues. STAT3 is a well-known transcription factor that regulates cell proliferation and promotes angiogenesis via upregulating VEGF in normal tissues. Nonetheless, STAT3 aberrant activity has been linked to cancer progression and metastasis by promoting the expression of pro-angiogenic factors such as VEGF-A^62,63^. As a result, several oncogenic targets of STAT3, including VEGF, have been detected, and inhibitors of STAT3 have been developed as novel treatment strategies for cancer^63,64^. Additionally, STAT3 induces the expression of hypoxia-inducible factor-1α (HIF-1α), which is another crucial mediator of angiogenesis. The simultaneous binding of both STAT3 and HIF-1α to the VEGF-A promoter leads to the maximum transcriptional activation and subsequent promotion of angiogenesis^63^. The other transcription factor, E2F1, is a member of the E2F family of transcription factors and has been implicated in cellular proliferation, differentiation, and apoptosis in colon cancer cells. It was recently found that E2F1 also participates in the metastasis and chemoresistance of colon cancer^65^. The other experimentally validated transcription factor, Sp1, is also shown to increase the expression of VEGF-A and VEGFR^66^. Future studies may investigate the role of these TFs in EMAST^+^ phenotype in CRC patients and also delve into the underlying involved signaling pathways to develop new therapeutic targets.

Aberrant angiogenesis not only provides tumor cells with nutrient and oxygen, but it is also an effective barrier against the host immune system. Therefore, we also investigated the correlation of ANGPT-1, ANGPT-2, and VEGF-A genes with immune gene signatures and found significant association of ANGPT-1 and VEGF-A expressions with most of the lymphocytes and immune system regulators, especially in COAD tissues (Supplementary 1) (http://cis.hku.hk/TISIDB/). These immunosuppressive effects by tumor cells have been a target of recent treatment strategies to circumvent immunosuppressive tumor microenvironment^67,68^. The tumor microenvironment functions as an intense barrier, primarily due to the disrupting impacts of irregularly patterned and tortuous tumor vessels on immune cells, leading to tumor progression and potentially facilitating tumor resistance to cancer therapies^69^. The disorganized vascular network within tumors significantly inhibits the infiltration of CD8^+^ T cells into the tumor microenvironment, thereby compromising their efficacy^70,71^. Additionally, in tumor-bearing hosts, VEGF-A can modulate the behavior of other immune cell populations, including regulatory T cells, myeloid-derived suppressor cells, and tumor-associated macrophages, consequently giving rise to the development of tumor-promoting (immunosuppressive) M2-like macrophages^72,73^. Hence, angiogenesis and immunosuppression are closely associated and occur simultaneously in response to stimuli. As shown in this study, ANGPT1 and VEGF-A are closely associated with EMAST^+^ phenotype of CRC, and therefore, experimentally investigating the association between EMAST^+^ phenotype with immune signatures can be of paramount importance. Combining anti-angiogenic therapy with immunotherapy may demonstrate a synergistic effect on EMAST^+^ tumor suppressive strategies.

At the moment, the most frequently clinically approved anti-angiogenesis drugs are VEGF inhibitors, such as Bevacizumab. Albeit, anti-VEGF drugs confront certain limitations, such as low progression-free survival (PFS) and tumor resistance^13^. Moreover, it is essential to consider that multiple pathways are responsible for regulating tumor angiogenesis, and in cases where one pathway is obstructed, alternative pathways may compensate. This highlights the significance of targeting additional angiogenic pathways, such as angiopoietins^74^. In this regard, Trebananib is a peptide inhibitor that neutralizes both ANGPT-1 and ANGPT-2 interaction with the Tie-2 receptor, reducing tumor angiogenesis^75^. In order to evaluate the effectiveness and safety of the Bevacizumab and Trebananib combination therapy in the absence of chemotherapy, researchers conducted a first-line treatment study targeting metastatic colorectal cancer. This study revealed satisfactory efficacy of the dual anti-angiogenic combination therapy, as evidenced by the occurrence of durable responses^76^. Moreover, ongoing research is being conducted on Nesvacumab and MEDI-3617, which are high-affinity monoclonal antibodies functioning as anti-ANGPT-2 agents^77–79^ (Supplementary File 1; see https://clinicaltrials.gov/ and https://www.dgidb.org/ for more details). At present, the combination of anti-angiogenesis therapy with chemotherapy, targeted therapy, or immunotherapy has been approved for clinical application and has dramatically improved the survival rates of cancer patients^75,80^.

In summary, the data gathered from the current study suggests that angiogenesis-related genes, specifically ANGPT-1 and VEGF-A, may play a significant role in increased metastasis and relatively poorer survival outcomes observed in EMAST^+^ colorectal cancer. These findings highlight the potential importance of targeting angiogenic pathways in the management of EMAST^+^ colorectal cancer to improve patient outcomes. Furthermore, EMAST biomarker can be referred to as an additional aspect of Consensus molecular subtype 4 (CMS4) biology, due to the fact that CMS4 tumors display activated angiogenesis and worse overall survival. The Consensus molecular subtype (CMS) classification of CRC currently classifies colon tumors into four subclasses by gene expression profiles, and among them, CMS4 is more likely to form metastasis^81^. It is suggested that a combination of factors used in this new classification system, including angiogenesis-related ones, can have superior predictive value and play a fundamental role in the metastatic dissemination of CMS4 tumors. Further research is required to deeply determine the functional mechanisms of ANGPT-1, ANGPT-2, and VEGF-A in EMAST^+^ CRC progress and metastasis.

## Conclusions

In conclusion, we identified a significant increased VEGF-A and decreased ANGPT-1 expressions in EMAST^+^ colorectal tumors in CRC patients. Moreover, our study identified a significant association between VEGF-A and ANGPT-2 overexpression, as well as ANGPT-1 downregulation with colorectal tumor metastasis and reduced overall survival. Through in-silico analysis, we expanded our study by constructing regulatory networks that encompassed these angiogenic genes in colorectal cancer. Nevertheless, it is crucial to acknowledge that the generalizability of these findings to EMAST^+^ colorectal cancer requires further experimental investigations. Based on accumulating data from the present study, it is proposed that EMAST biomarker can be referred to as an additional aspect of CMS4 biology. Since CMS4 tumors display activated angiogenesis and worse overall survival, the results of this study may be beneficial to guide personalized treatments.

### Supplementary Information

Supplementary Figures.

Supplementary Information 2.

## Data Availability

The experimental data that support the findings of this study are not openly available due to reasons of sensitivity and are available from the corresponding author upon reasonable request. The in-silico analyses conducted in this study, as well as the datasets presented, are accessible through online repositories. The names of these repositories, along with the accession numbers, were provided in the article.

## Abbreviations

AUC: Area under curve
ANGPT1: Angiopoietin-1
ANGPT2: Angiopoietin-2
CeRNA: Competitive endogenous RNA
CircRNA: Circular RNA
CMS: Consensus molecular subtype
COAD: Colon adenocarcinoma
CRC: Colorectal cancer
DEG: Differentially expressed gene
EMAST: Elevated microsatellite alteration at selected tetranucleotide repeats
EMT: Epithelial-to-mesenchymal transition
GEO: Gene expression omnibus
GENT2: Gene expression database of normal and tumor tissues 2
LncRNA: Long noncoding RNA
MiRNA: Micro RNA
MMR: Mismatch repair
MSI: Microsatellite instability
NAT: Normal adjacent tissues
PPI: Protein–protein interaction
READ: Rectum adenocarcinoma
RQ: Relative quantification
TF: Transcription factor
VEGF: Vascular endothelial growth factor
VEGFR: Vascular endothelial growth factor receptors
