# Supplementary material for: Differential expression of angiogenesis-related genes ‘VEGF’ and ‘angiopoietin-1’ in metastatic and EMAST-positive colorectal cancer patients
Source: Sci Rep. 2024 May 8;14:10539. doi: 10.1038/s41598-024-61000-x (PMC11079037; doi:10.1038/s41598-024-61000-x)
Supplement: Supplementary file 1 — Supplementary Figures. [file 41598_2024_61000_MOESM1_ESM.docx]

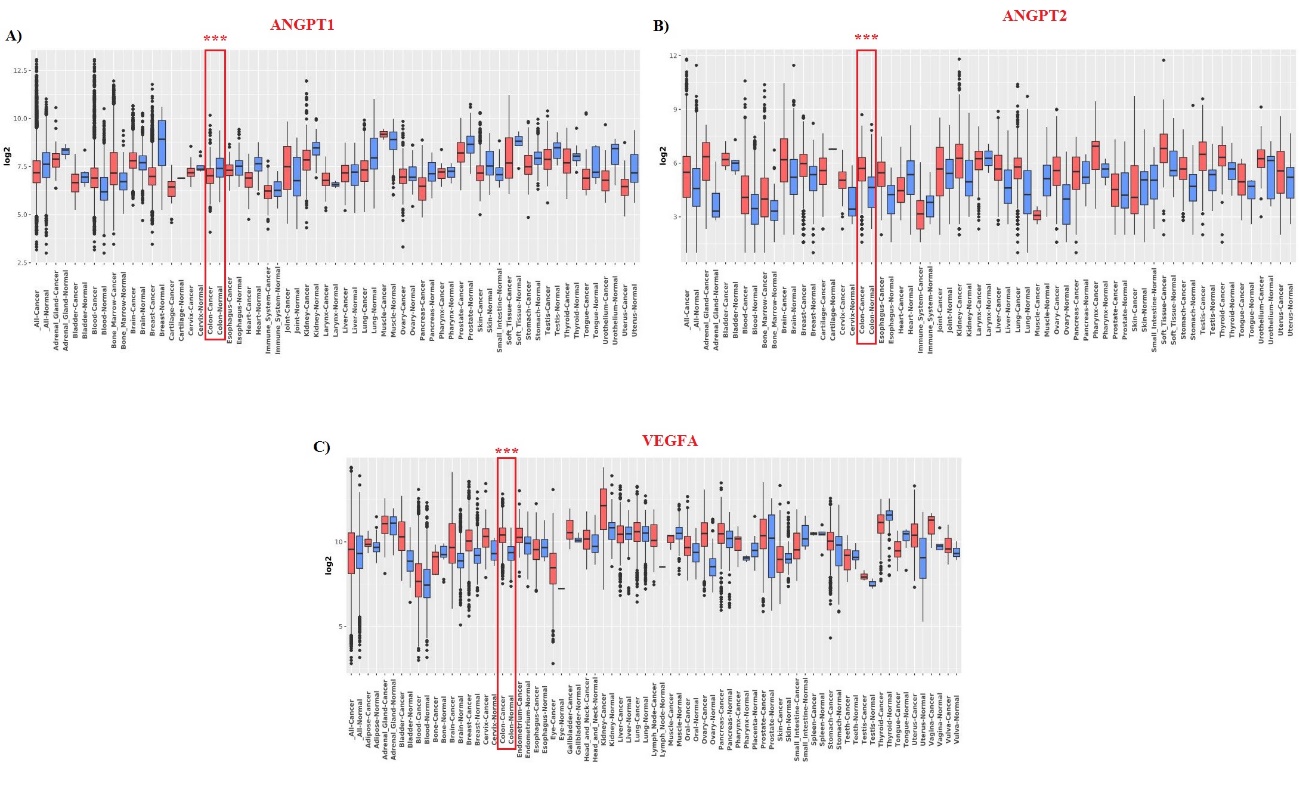


**Supplementary Figure 1.** **Gene expression profiles of ANGPT1, ANGPT2, and VEGFA based on microarray obtained from GENT2 database.** **(A**) ANGPT-1 is down-regulated in COAD and READ, however, ANGPT-2 **(B)** and VEGF-A **(C)** were upregulated in COAD and READ compared to normal tissues.

**
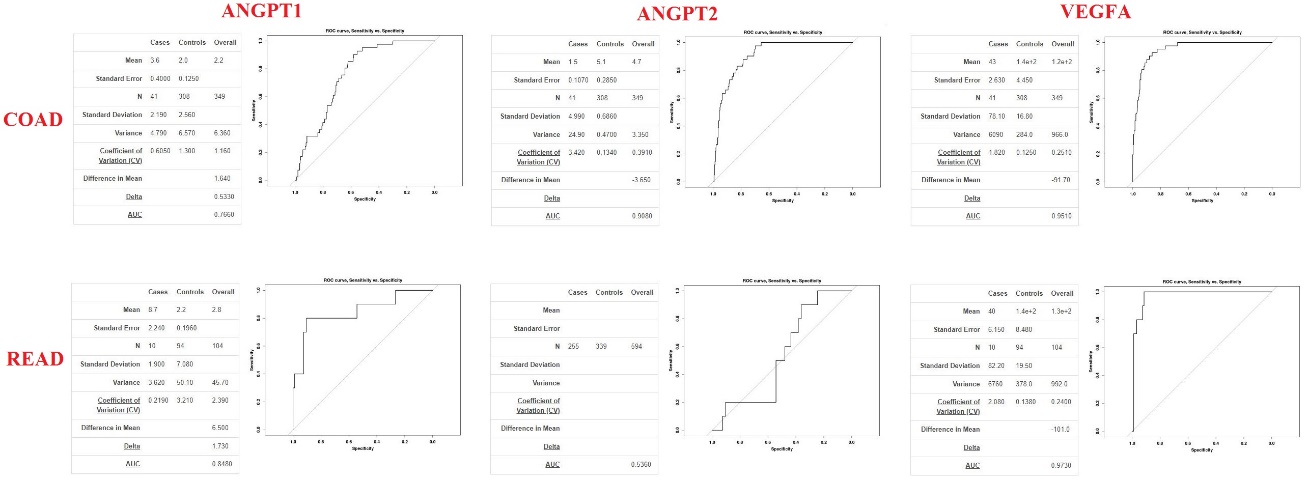
**

**Supplementary Figure 2.** **Diagnostic values of ANGPT-1, ANGPT-2, and VEGF-A in COAD and READ**. ANGPT-2 and VEGF-A in COAD and VEGF-A in READ had AUC >90%.


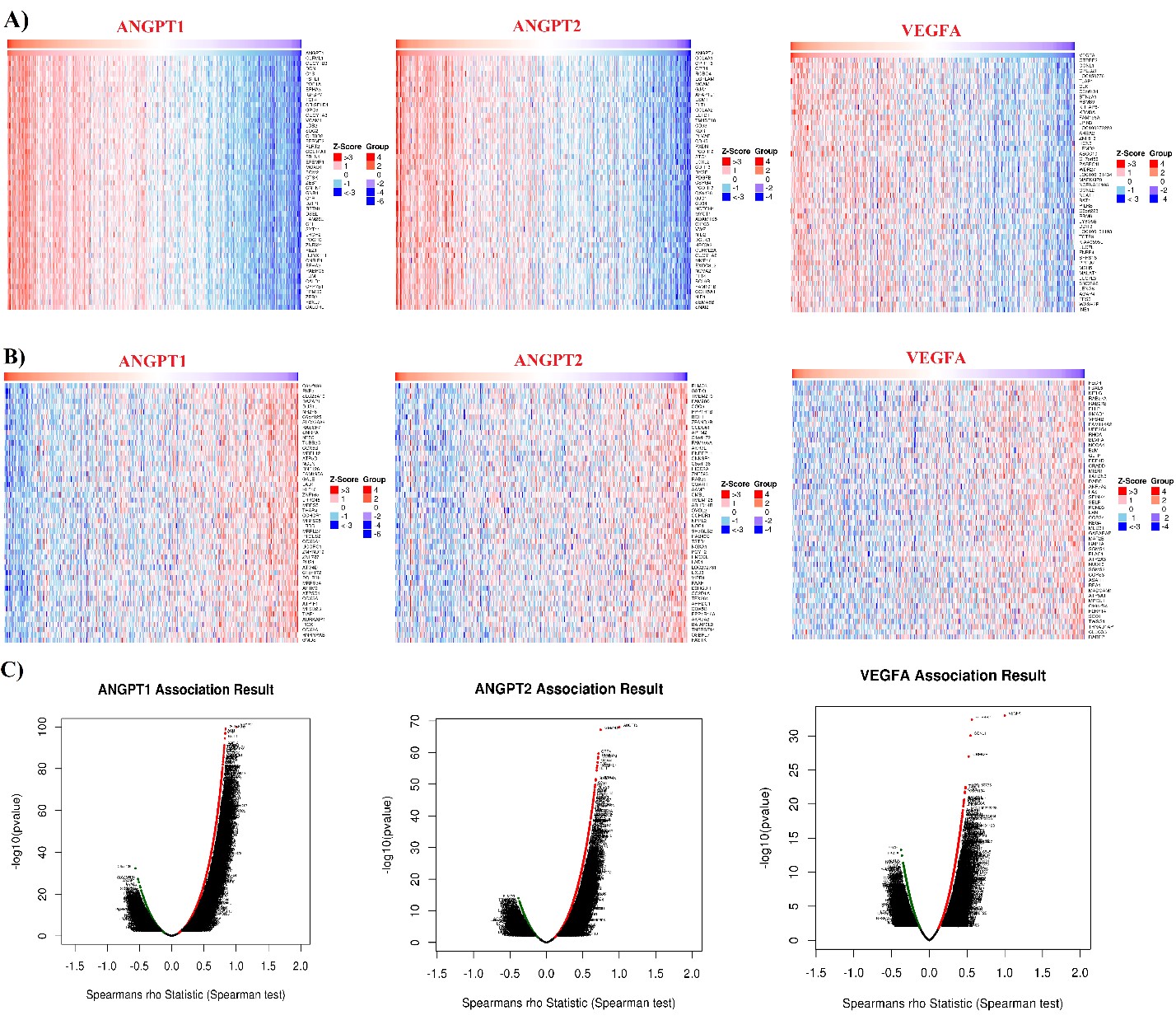


**Supplementary Figure 3.** **Co-expression network of ANGPT-1, ANGPT-2, and VEGF-A.** Top 50 positively **(A)**, 50 negatively **(B)**, and volcano plot of the genes positively and negatively correlated with ANGPT-1, ANGPT-2, and VEGF-A**(C)**.

**
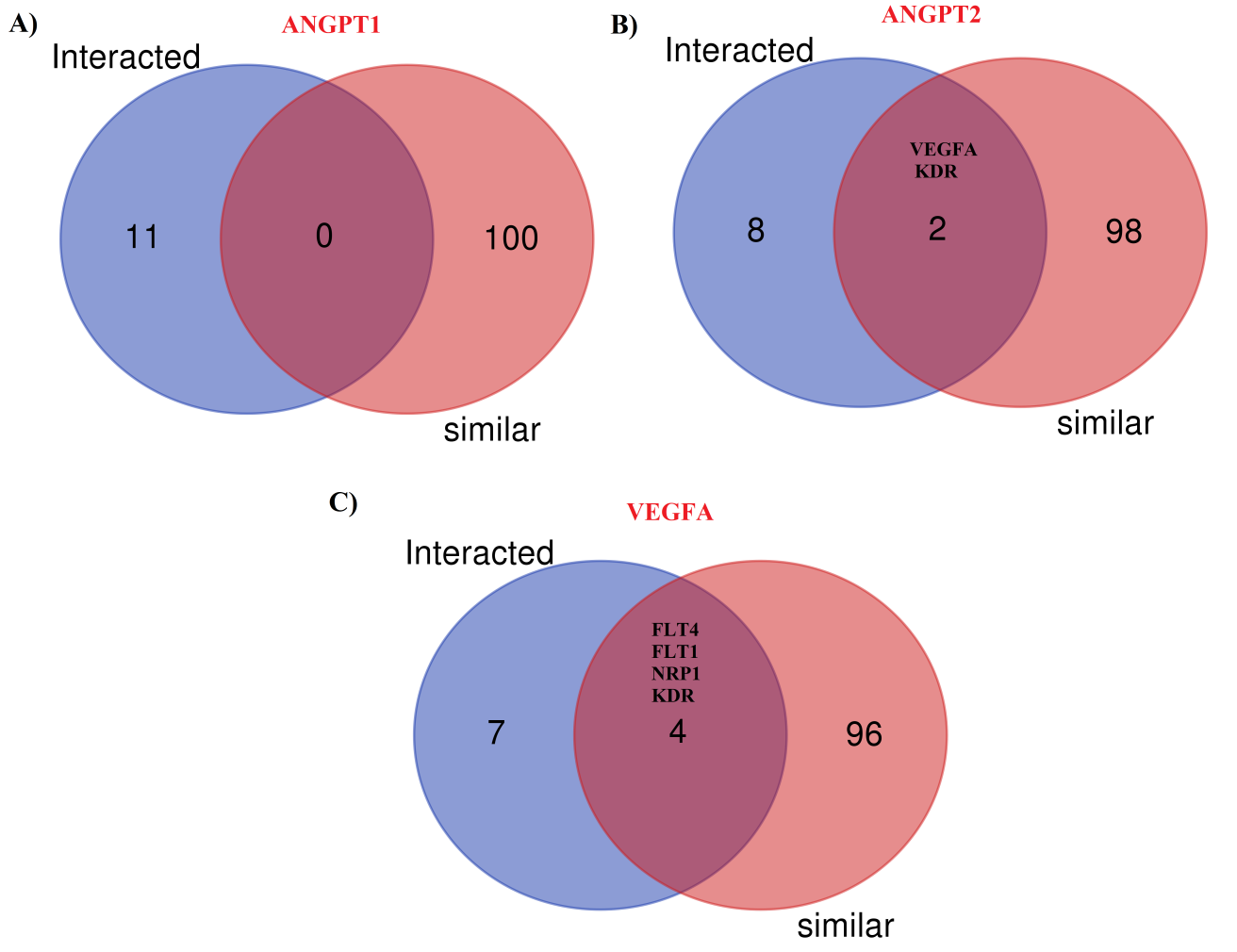
**

**Supplementary Figure 4.** **Intersection analysis of ANGPT-1, ANGPT-2, and VEGF-A similar and interacted genes.**


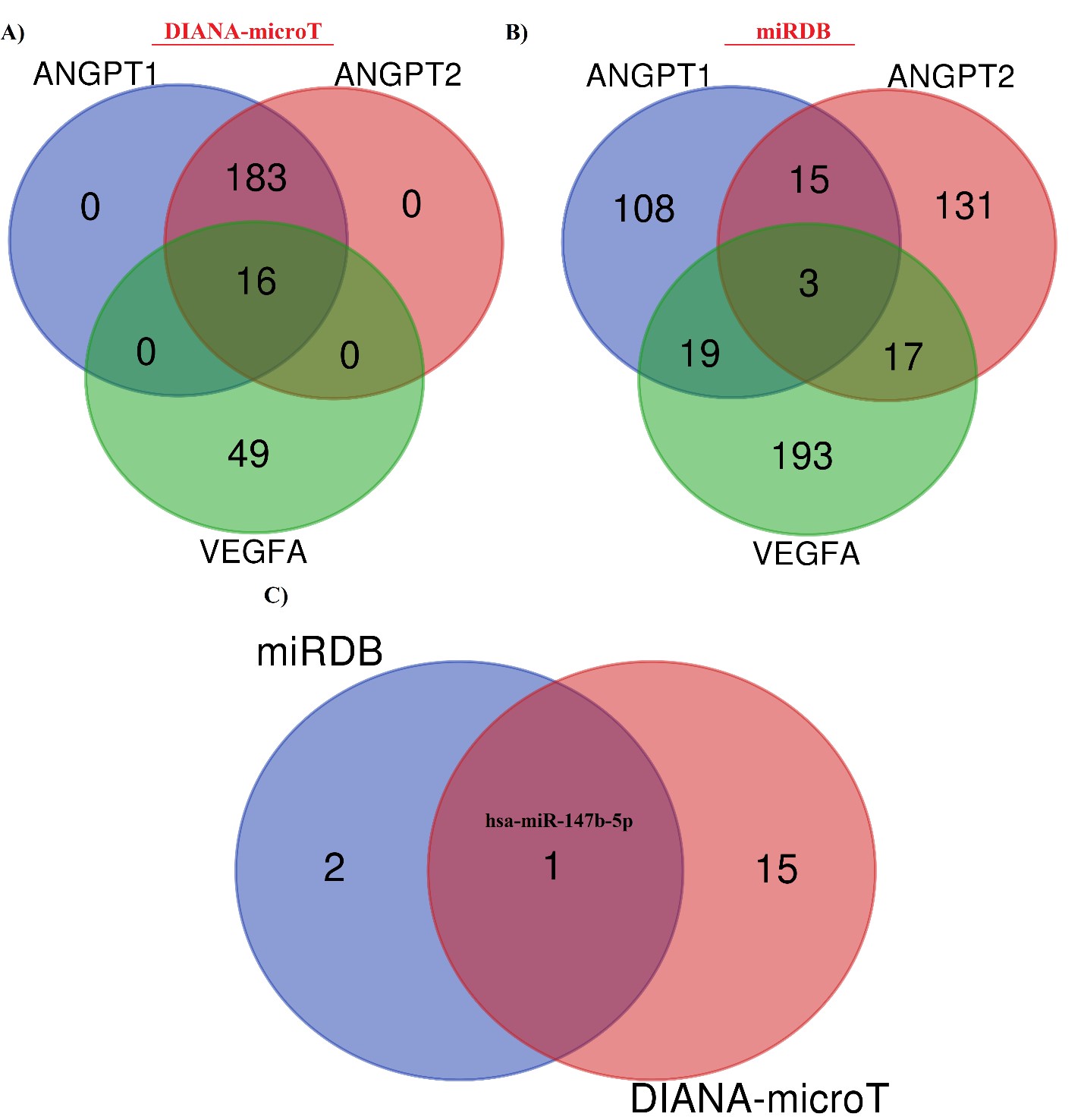


**Supplementary Figure 5.** **miRNAs targeting ANGPT1, ANGPT2, and VEGFA.** A total of 16 and 3 miRNAs were found targeting three selected genes based on DIANA-microT **(A)** and miRDB **(B)**. By intersectional analysis, *hsa-miR-147b-5p* was found as a common miRNA targeting three genes based on both miRDB and DIANA-microT **(C).**


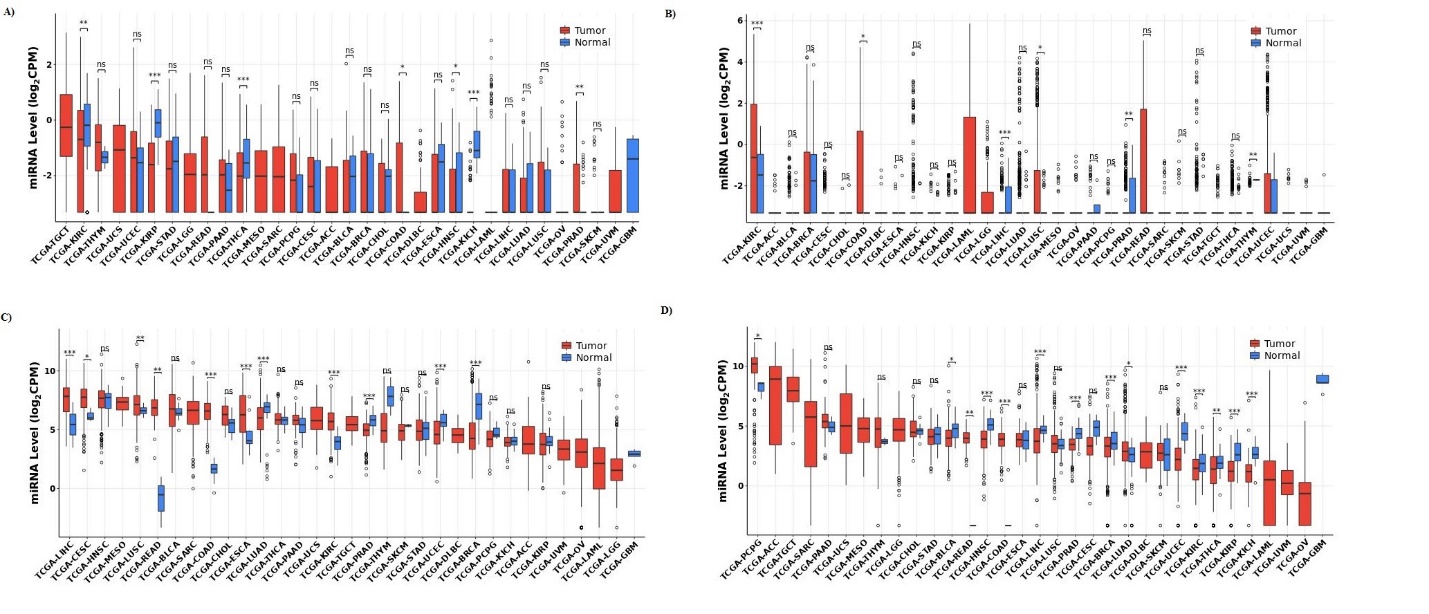


**Supplementary Figure 6.** **Experimentally validated miRNAs in TCGA COAD or READ among identified miRNAs.** hsa-miR-190a-3p **(A)**, hsa-miR-374c-5p **(B)**, hsa-miR-452-5p **(C)**, and hsa-miR-889-3p **(D)** were experimentally validated in TCGA COAD or READ tissues based on CancerMIRNome database.


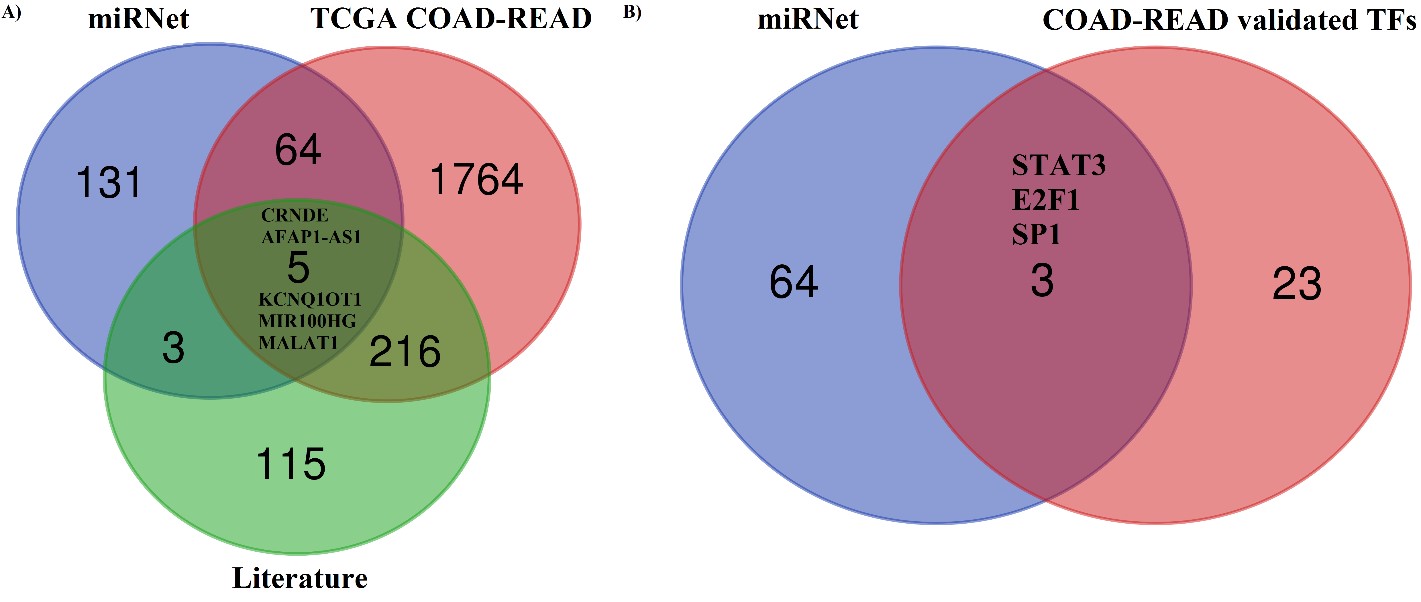


**Supplementary Figure 7.** **lncRNAs of identified miRNAs and TFs of ANGPT1, ANGPT2, and VEGFA.** **(A)** Five lncRNAs including CRNDE, AFAP1-AS1, KCNQ1OT1, MIR100HG, and MALAT1 were experimentally validated as a differentially expressed lncRNAs either in TCGA COAD or READ and literatures based on LncTarD 2.0. **(B)** Among 67 TFs identified based on miRNet, STAT3, E2F1, and Sp1 were experimentally validated in COAD-READ tissues based on TFcancer database.


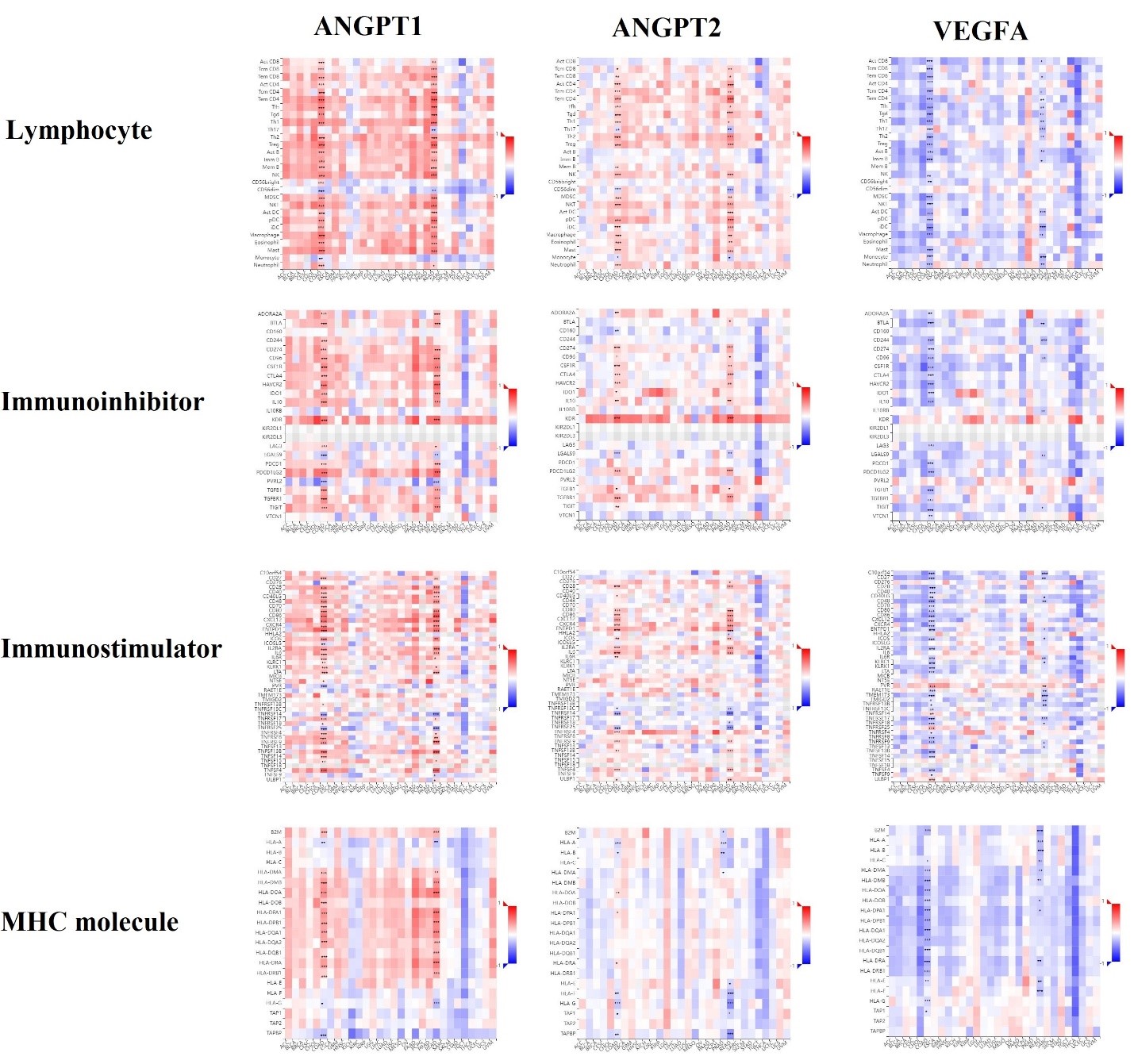


**Supplementary Figure 8. Correlations between ANGPT-1, ANGPT-2, and VEFG-A and immune gene signatures based on TISIDB database.** There were significant correlations between ANGPT-1 and VEGF-A expressions and most of the lymphocytes alongside immunoinhibitors, immunostimulator, and MHC molecules, especially in COAD tissues


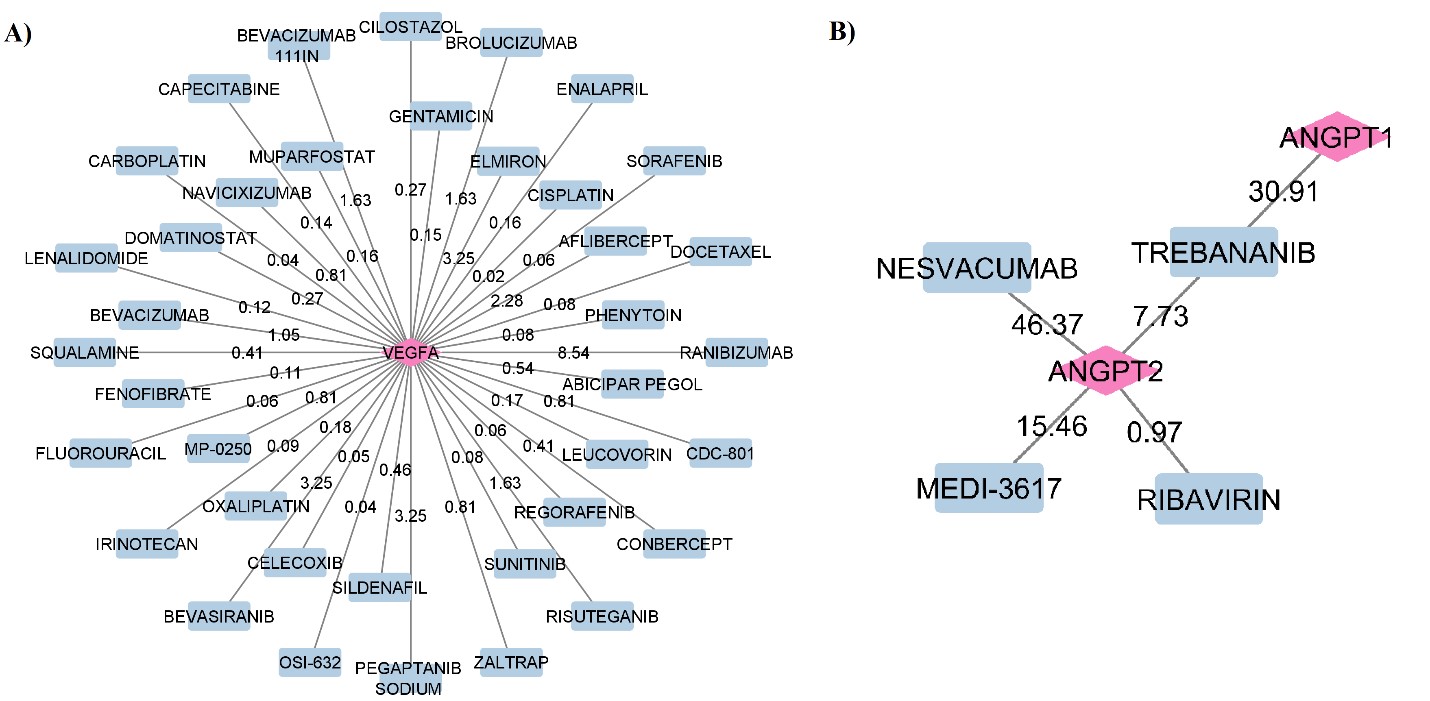


**Supplementary Figure 9. Candidate drugs target ANGPT-1, ANGPT-2, and VEGF-A. (A)** Thirty-eight drugs were found to have an interaction with VEGF-A. **(B)** One and four drugs were found to have interaction with ANGPT-1 and ANGPT-2. Interaction scores were recorded on the edges
